# The usability of Jordan stillbirths and neonatal deaths surveillance (JSANDS) system: results of focus group discussions

**DOI:** 10.1186/s13690-021-00551-1

**Published:** 2021-03-07

**Authors:** Yousef S. Khader, Khulood K. Shattnawi, Nihaya Al-Sheyab, Mohammad Alyahya, Anwar Batieha

**Affiliations:** 1 Epidemiology, Medical Education and Biostatistics, Department of Community Medicine, Public Health and Family Medicine/ Faculty of Medicine, Jordan University of Science & Technology, Irbid, 22110 Jordan; 2 Maternal & Child Health Nursing Department, Faculty of Nursing, Jordan University of Science and Technology, P.O.Box (3030), Irbid, 22110 Jordan; 3 Child and Adolescent Health, Allied Medical Sciences Department, Faculty of Applied Medical Sciences, Adjunct professor at the Faculty of Nursing, Jordan University of Science and Technology, P.O.Box (3030), Irbid, 22110 Jordan; 4 Health Management and Policy, Department of Health Management and Policy, Faculty of Medicine, Jordan University of Science and Technology, P.O.Box (3030), Irbid, 22110 Jordan; 5 Department of Public Health, Jordan University of Science and Technology, Irbid, Jordan

**Keywords:** Stillbirths, Neonatal deaths, surveillance

## Abstract

**Background:**

Jordan Stillbirths and Neonatal Deaths Surveillance system (JSANDS) is a newly developed system and is currently implemented in five large hospitals in Jordan. This study aimed at exploring the healthcare professionals’ perception about the usability of JSANDS.

**Methods:**

A descriptive qualitative approach, using focus group discussions, was adopted. A total of 5 focus groups including 23 focal points were conducted in five participating hospitals in Jordan.

**Results:**

Data analysis identified nine main issues related to the JSANDS system: the system usefulness, the system performance, data quality, the system limitations, human rights, female empowerment, nurses’ competencies strengthened, the sustainability of the JSANDS, and COVID-19 impact on the system. Users reported that JSANDS data were useful, the system was simple and easy to use, and the data were accurate and complete. However, some users reported that some technical issues need to be enhanced.

**Conclusions:**

JSANDS was perceived positively by the current users. According to them, it provides a formative and comprehensive data on stillbirths and neonatal deaths and their causes, and therefore, was recommended to be adopted by its users and scaled up.

**Supplementary Information:**

The online version contains supplementary material available at 10.1186/s13690-021-00551-1.

## Background

In many countries of the world, the majority of stillbirths and part of neonatal deaths are not registered [1]. Therfore, there is a need to improve and strengthen the registration systems of stillbirths and neonatal deaths around the world to accurately estimate these deaths and their causes. This is important for reducing such deaths and ensuring human rights [1].

Health data are the essence of any health care system as such data form the basis for the development of evidence-based practices and making important medical and policy decisions. Jordan has limited data system documenting and reporting stillbirths and neonatal deaths and their causes. In addition, the existing sources of data on stillbirths and neonatal deaths are liable to biases. Therefore, improving a reporting system of stillbirth and neonatal deaths is critical for tracking progress and taking appropriate actions. An electronic Jordan Stillbirths and Neonatal Deaths Surveillance system (JSANDS) was developed and implemnted in five large hospitals in Jordan since August 2019. JSANDS was developed as a secure on-line data entry system to collect, organize, analyse, and disseminate reliable data on stillbirths, neonatal deaths, and their causes. The JSANDS system collects comprehensive individual-level data on each death that allows for better adjustment of the crude mortality rates, allows for detailed exploration of the causes of perinatal mortality in Jordan, calculates and reports indicators and provides a clearer insight into the risk factors most commonly associated with stillbirth or neonatal death. The complete description of JSANDS has been reported in a previous study [2]. As of 1 September 2020, a total of 23,758 births were registered and reported.

Evaluation of the technical factors of the system cannot merely determine its success [3]. Feedback from the current JSANDS users is an important step to further improve the system before it becomes fully implemented in all Jordanian hospitals. Therefore, there is a need to consider other factors in evaluation such as social influences and users’ perception. This study aimed at exploring the healthcare professionals’ perception about usability of the newly implemented JSANDS system in five pilot hospitals.

## Methods

A descriptive qualitative approach, using focus group (FG) discussions, was conducted after one year of the JSANDS’s implementation in 5 pilot large hospitals. Three of these hospitals were public Ministry of Health hospitals, one is a university hospital and one is a private hospital. A purposive sample of focal persons who had previously used or were still using the JSANDS system was invited to participate in this study. The focal points in each hospital acted as a contact key between the relevant hospital and the JSANDS team and they were responsible for documenting the deliveries, training other healthcare professionals (HCPs) on using the system, and monitoring the documentation process. Five FG discussions were conducted with the focal points; one in each hospital. The number of participants in each FG ranged from 4 to 5 and the total number of participants was 23 HCPs.

FG discussions took place in a quiet meeting room in each hospital and lasted between 60 and 80 min in duration. To maintain consistency, the same moderator facilitated all five FGs. Participants were asked to respond to the main open-ended question, “How would you describe your experience with using the JSANDS system?”. Follow-up questions were asked when clarification was needed. Interview guide (Appendix 1) was developed by the researchers and used to moderate the FG discussions. Questions were sequentially asked in each FG allowing appropriate time for a discussion of each question. The moderator asked questions and guided the group discussion, and a second researcher observed the conversation and took notes. Both, the moderator and the researcher were female researchers holding PhD degrees in nursing. FG discussions were held at a convenient time to all participants, and were audio recorded with permission.

The ethical approval was obtained from the Institutional Review Board at Jordan University of Science and Technology. Participation in the FG discussions was voluntary and participants were informed that they have the right to withdraw at any point.

Deductive content analysis, also called a directed content analysis, approach was adopted in this study, in which analysis was based on predetermined questions that needed to be answered by the participants [4]. This approach can help focus on the research questions and can provide predictions about the study variables, and therefore, help in determining the initial coding and relationships between codes [4]. According to Elo & Kyngäs, this approach can be used when a researcher has some idea about the responses from the participants [5]. After transcribing the FG discussions, data analysis began by identifying key concepts as initial coding categories. The interview questions were used as a guide to data analysis. Researchers identified all examples of a particular predetermined code. Coded data were then categorized into themes and subthemes.

## Results

Five FG discussions were conducted with 23 focal points who had previously used, or were still using the JSANDS system. All participants were females and were either nurses or midwives. Their age ranged from 25 to 50 year and their years of experience ranged from 3 to 28 year. The focal points consisted of senior head nurses of labour and NICU departments, and senior staff nurses and midwives who were using the system since its implementation.

Data analysis identified nine main issues related to the JSANDS system (Table 1): 1. System usefulness, 2. System performance, 3. Data quality, 4. The system limitations, 5. Human rights, 6. Female empowerment, 7. Nurses’ strengthened competencies, 8. Sustainability of the JSANDS, and 9. COVID-19 impact on the system.

**Table 1:** The study themes and subthemes

Themes	Subthemes
1. System usefulness	• Detecting possible risk factors
	• Care improvement
	• Identifying marginalized population and misfortune areas
	• Continuing educational opportunities
2. System performance	• Ease of use
	• Cost-effectiveness
	• Privacy and security
3. Data quality	• Completeness
	• Accuracy
4. Human rights	• Human rights
5. Empowerment of female nurses	• Knowledge is a treasure
6. Health professionals’ competencies strengthened	• Improved work • Promoted communication skills
7. Sustainability of JSANDS	
8. COVID-19 impact	
9. Limitation of the system	• Technical problems
	• Possible unauthorized access
	• Issues with reporting the cause of death

### The system’s usefulness

All participants in the 5 FGs agreed on the usefulness of the data collected by the system and indicators generated by the system, and gave many examples of how these data could be useful. One participant talked about how the data opened an eye on many possible risk factors of stillbirths and neonatal deaths:

*There were instances of stillbirths or neonatal deaths that went unnoticed. When we looked at the data generated by the system, we started to notice patterns and clusters of possible causes of neonatal deaths. Maternal preeclampsia cases, for example, used to go unrecognized, which turns out to be a cause of neonatal deaths.*

The participants talked about the significant amount of possibilities of using the system and the data generated by it, which might help in the improvement of care delivered to patients and their families:

*We can now know how many births, and how many deaths we had over a period of time. We also can judge our quality of care based on these deaths. Our attention to certain causes of neonatal deaths led us to start giving health education classes for mothers immediately after birth. A good feature of it is that you can know if a woman has had previous delivery in our hospital and so as to retrieve all her information in a click of a button.*

The participants stated that the system allowed them to see trends and patterns of basic statistics of all deliveries and births that occurred in their hospitals over a period of time, and to compare these data with the other hospitals using the system.

*Data generated from JSANDS are very helpful to us. We can know the number of deliveries we had for any period of time with a click of the button. We also can look at the percentage of caesarean sections for example and compare our performance to other hospitals.*

Participants talked about how accurate was the data generated by the system and described instances on how did the system detect a significant and unusual increase in the number of neonatal deaths and stillbirths. This finding might help to raise the attention of healthcare professionals to promptly investigate the causes of this problem and to take actions. These actions included modification of some hospital policies, staff education and training, and mothers’ education during antenatal visits.

*Based on the JSANDS data we noticed that the majority of the deaths occurred as a result of a defect in antenatal care. So we thought that teaching mothers during this period is the basis for any change. Therefore, we gave continuing education classes for our staff and we trained up to 80% of them to be able to educate mothers.*

Participants reported that the system also helped in identifying marginalized population and misfortunate areas:

*We noticed that many of the deliveries that ended with a stillbirth or neonatal death were coming from “Alghowr”. The long distance to the hospital was mainly a reason for them to arrive late. Now, we are given those people who come from this area more attention.*

One added:

*The system succeeded in highlighting some marginalized people and identified disadvantages areas in Mafraq city. Many women with complications were coming from these areas. Identifying these disadvantaged areas helped us in providing better care for them*

According to the participants, JSANDS helped in identifying high risk population such as pregnant women with diabetes or preeclampsia. This has an important value as it helps predict possible complications. One participant commented on this “*It helped us in identifying high-risk women such as women with diabetes and women with preeclampsia.. These women are more susceptible to complications”.*

Other participant commented on how the data generated by the system gave them the chance for more continuing educational opportunities:

*It is a good experience. It gave us a lot of information that made us focusing on things that we did not focus on before. I mean, the hospital benefited from the system greatly. It directed our attention to the need for educating the health care team and nurses about issues related to neonatal and maternal care. It was the drive for refreshing our knowledge about certain issues related to child and mother health.*

### The system’s performance

JSANDS users had provided feedback relating to the system features. They identified many features that they liked about the system including the ease of use, privacy and security, and reported some limitation of the system. Users reported that the system was simple and easy to use and it was easy to train others on the use of the system. One focal point said, “*It is very easy and straightforward. I mean, it does not take much time to fill out all the information”*. Another added “*Not much effort was made when we trained the rest of our staff. The guidelines are easy and take no time to follow*”. One of the focal points described the system as:

*One can modify, amend or change any data already filled when it is needed. The identification of any case make it also flexible. I mean you only have to enter the mother’s name or her national ID number and the system retrieves all of her information; you don’t need to re-enter all her information again.*

Participants thought that the system is cost-effective, and therefore, they recommended to implement it in all hospitals across the country “The *system is cost-effective and its implementation in other hospitals requires little cost”.*

### Data quality

Participants discussed the quality of data that are generated by the system and described issues related to the completeness, sensitivity and accuracy of the data. They liked the feature in the system that that does not allow them to save and store any case data with any missing information, and this confirms, according to them, the completeness of the data generated by the system:

*The information entered is 100% correct and complete because there is always someone responsible for monitoring the entries on a daily basis to ensure that all cases have been entered. Also, the entries cannot be saved unless the case information is complete.*

Because of the usefulness, completeness, and accuracy of the JSANDS data, the participants agreed on the necessity of the adoption JSANDS as an electronic stillbirths and neonatal deaths surveillance system by the Ministry of Health (MOH) and to be implemented in all hospitals across the country. One participant said:

*I highly recommend adopting this system. If adopted by, we can easily retrieve any information about any patient, who has had a previous admission in any hospital across the country. It is important to receive all the details of the patient’s medical history, which will be reflected in the level of service provided to this patient*

### Human rights

Participants discussed issues related to the system consideration of human rights in general and children’s rights in particular. The participants described the system as generally secure with good privacy. They stated that JSANDS protects patient privacy to a high degree. One participant said “*In general, the system is safe, and each one of us has a user password, and there is a difference between what information each one of us is allowed to see depending on our roles”.*

According to the participants, the JSANDS has also acknowledged children’s rights by reporting stillbirths:

*Before we started with the JSANDS, stillbirths were not registered at all. They were treated as if they were nothing or non-existent. They were not registered in the family book and no birth certificate was issued for them. Thanks to JSANDS for counting all babies*

They agreed and stressed on the need to register every single death, “*every child has the right to be registered regardless of its status and birth outcome”.*

### Empowerment of female nurses

The bulk of information and knowledge that the system offers has an impact on the empowerment of female nurses as, according to them, JSANDS gave them the power to stand in front of other health care team, mainly physicians, and have a good discussion about the cases:

*The knowledge we get gives us power. As a female nurse, we started to have discussions with the doctors during our meeting in the death review committee. They’re listening to us now and value what we have to say about the cases, as we are the ones who meet with the mothers and fill out the forms.*

Another participant described the information received from the JSANDS as a treasure that gave her a power to engage in any health related discussion:

*The information that we have from JSANDS is a treasure. This treasure of knowledge gives me power, which enhanced my confidence to engage in any discussion with the other health care team.*

### Health professionals’ competencies strengthened

All participants were satisfied with JSANDS. They described how the system helped them to improve their work and enhance their communication skills with the families and how it changed the way they currently perceive stillbirths and neonatal deaths.

Having this system in their hospitals made them curious about getting some statistics, which changed their perception about some facts:

*When we started using JSANDS, we noticed that the caesarean section (CS) rate is much higher than we expected. For example, that last 12 cases we had, 9 of them were CS. Frankly, when I saw this piece of information I was shocked by the rate. We don’t usually look for such statistics. This made me think that the current practice is not right and it has to be changed*

Another participant talked about how JSANDS changed the way she responds to some cases:

*What’s good about it is that when we have a woman, with a stillbirth for example, we can go back to JSANDS and retrieve her information and read her history. We see how many times she has had stillbirths and we communicate this with her doctor and encourage the doctor to do more investigation.*

Participants also believed that JSANDS changed the way that the health care providers perceive the deaths and how this enhanced their sense of responsibility:

*It enhanced the doctors’ accountability for their actions and care as they started focusing on the number of stillbirths and deaths that are registered under their names, and they become afraid of being labelled of having too many deaths during their shifts. This will certainly make them think more about the causes and to try their best in finding solutions.*

Participants described how JSANDS promoted their communication skills with the families:

*JSANDS enhanced our communication skills with families. We talk to them more which strengthen our relationship with them. I mean, you know, we are not focusing only on numbers as we used to, but rather we started focusing on the quality of care and how to benefit the family most, and not to allow them to experience any death again*

### Sustainability of JSANDS

Participants made several suggestions to improve and sustain the system. For instance, some participants suggested to integrate the JSANDS with other health information systems already employed in their hospitals. Many participants recommended integrating the JSANDS into an existing electronic health information system called Hakeem “*It became more helpful if this system is combined with Hakeem. This will give us a holistic kind of information.”*

Some participants believed that if they need to continue using the system, they “*must get incentives*”. One participant added “*Honestly, we need financial incentives because we are overwhelmed by the workload.”*

Although the system intended to capture only the most important variables related to deliveries in order not to overwhelm the users with too many entries, many users reported the need to add more information that was perceived as important in identifying high risks pregnancies such as information related to CS or NICU admission.

*When we enter the maternal causes for any stillbirth or neonatal death, the choices are according to the ICD10 are general. It would be helpful if you add a small box under our choice of the maternal cause to write what exactly happened to the patient with PIH for example*

### The impact of Covid-19

As the FG discussions were conducted during COVID-19 pandemic, we asked participants about the impact of COVID-19 on the birth and delivery process and on the number of stillbirths and neonatal deaths. As the system is designed not to allow users to modify entries after 28 days’ period for a security purposes, this was perceived as a limitation by the participant, especially because some of them were unable to work while in quarantine during the COVID-19 outbreak:

*The system is good and it has achieved its goals, but it does not allow us to modify entries after 28 days. We consider this as a limitation because during the quarantine while we were detained in our homes and returned back to work after a month or so, we couldn’t modify the previous entries. The developer need to consider this issue for similar emergency situations*

In addition, participants described situations in which the pandemic has both positive and negative impact on their work. On one hand, the outbreak impacted positively on the quality of care received by the women and their children, and on the other hand, impacted negatively on the women care seeking behaviours. One participant responded:

*It was a positive impact. Although the number of births is the same, the mothers and their infants received higher quality care because of the many precautions to limit the transmission of the virus. For us as a staff, we had to work harder to achieve this. Every woman has her own room, with no visitors and high quality of care.*

Another participant talked about the negative impact the COVID-19 has on care seeking behaviours of women:

*The pandemic affected negatively the time of seeking care, as we had many premature births as a result of that delay. We had higher premature deaths during the months of April and May because of the effect of the pandemic on seeking care.*

One participant suggested a negative financial impact of the pandemic on the families who lost their jobs during the quarantine, which negatively influenced the maternal health:

*I think another impact of the COVID is the financial impact. Many families lost their income because of the quarantine, which impacted the women health and we started to have more anaemic women*

### Limitation of the system

Although the participants liked the performance of the system, they identified some potential limitations of the JSANDS. They reported some technical problems, such as problems with the internet connection and occasional loss of previously stored data “*It happened to me twice to complete the data about a delivery and the next day it was gone. I think it might be a connection or a technical problem.”*

Some focal points expressed concerns about one limitation that may lead to unauthorized access to patient records as the screen does not shut down automatically when left without signing out “*but in case if one forgets to sign out, it does not shutdown automatically and the screen remains open which may allow others to access private information without permission.”*

Another limitation that was recognized by the participants was about selecting the main cause of death from a drop-down menu that contains causes of deaths based on the International Classification of Diseases-Perinatal Mortality (ICD-PM). When the drop-down menu contains options that they believed none of which quite fit, they have to choose “others”. The participants suggested to add a free-text box where they could type what they believe is the main cause of death:

*When we record the cause of death we need to choose specific causes of death. But sometimes we can’t find the main cause of death in the list and according to ICD-10, we need to choose “others” as a cause. So it would be good to give us a choice to type this in a free-text box. I mean after choosing “others” a box should be opened to give us the chance to type our findings*

## Discussion

This study evaluates a newly developed electronic stillbirths and neonatal deaths surveillance system (JSANDS) from the perspectives of its current users. The JSANDS is a software that was developed to register and report stillbirths and neonatal deaths and their causes in Jordan. This system has a quality improvement tool and generates useful indicators that enhance the quality of health care for mothers and their children.

Participants commented positively on the usefulness of the JSANDS. One of the most important features of any successful system is its usability. Usability is important because it enable users to achieve the system’s goals effectively. The improvement of the usability of any electronic health system leads to the improvement of health care delivery [6]. Usability of any system involves a set of evaluation methods to understand user experience in order to develop more useful products [6]. According to the International Organization for Standardization (ISO), usability involves many components and is defined as “*the extent to which a system, product or service can be used by specified users to achieve specified goals with effectiveness, efficiency and satisfaction in a specified context of use*” [7]. Nielsen described five quality components of usability namely (a) learnability: how easy is it to learn basic tasks; (b) efficiency: how quickly to perform tasks; (c) memorability: easy to remember tasks; (d) errors: having minimal errors; and (e) greater satisfaction [8]. These attributes were clearly suggested by the majority of the JSANDS users, which suggested a higher level of usability of the system. During the pilot phase of implementing the JSANDS, it was tested by its potential users by means of online questionnaire. The users rated the language used in the system as clear (85.2%), the sequence of input data as clear (95.1%), the system is easy to use (61%), and the system online design as good to excellent (83%) [2].

In addition, the JSANDS allows for enhancing female empowerment, and identifying marginalized and high risk population who may receive less effective healthcare. Identifying marginalized and high risk population, and misfortunate areas, allowed for the implementation of some strategies that addressed these liabilities. According to a recent literature review of a 32 studies, EHR has the ability to identify and predict outbreaks and high risk areas, which help in decision making and planning for outbreaks [9]. While it is crucial to introduce this surveillance system to health care facilities, consideration of ethical and cultural issues is important for effective implementation.

According to the participants, the greatest advantage of the JSANDS system is that it produces complete data on stillbirth and neonatal death rates, as well as, determine the causes of these deaths. This is important because one of the main goals of this surveillance system is to reliably identify causes of stillbirths and neonatal deaths, which will aid in describing changes in mortality and identifying health care priorities in Jordan. This is essential for the development of health policies and programmes for preventing such causes of deaths.

The completeness of data entries and the accurate identification of the causes of stillbirths and neonatal deaths are important features of any surveillance system [10]. One of the main features of the JSANDS that promotes completeness of data is the presence of mandatory fields that obliges users to complete all required data, otherwise it will not be saved to the system. This feature offered by electronic health records was recognized by others to be significant and enhance patient safety [11, 12]. In addition, the majority of our participants discussed the need to integrate the JSANDS with other electronic health systems in their hospitals and to connect their hospitals with the primary health care settings, which allow for more comprehensive data and complete access to previous patient records. Opening lines of communication between health provider organizations is critical to the success of any electronic health system [13].

Although all details of newborn babies are recorded in the JSANDS to allow for comprehensive stored data, the focus groups’ participants recommended more details of the mothers to be added to the system in order to have a comprehensive linkage of data between mothers and their babies. This is in alignment with the WHO standards for improving quality of maternal and newborn care, where the quality statement 2.1 stated that “*every woman and newborn has a complete, accurate, standardized medical record during labour, childbirth and early postnatal period*” [14]. This is crucial for the early detection of complications and improved health outcomes [14].

There was an agreement between participants on the necessity of the adoption of JSANDS. According to them, the JSANDS has a number of features that make this system worth the adoption by the health care organizations. The positive attitude of users was related to many factors including their perceived usefulness of the data, ease of use, ease of learning, and the flexibility of the system. Adding to that, its ability to enhance human rights, identify the marginalized populations and misfortunate areas, and enhance female empowerment. Moreover, its positive impact on their work. A systematic review of the literature has identified similar factors that influence positive attitudes toward the adoption of electronic health records including, for example, the usefulness of data gathered via the system, the quality of information generated by the system, ease of access, reliability, and the speed of the system [15]. O’Donnell et al. has also described users’ characteristics that possibly associated with positive attitudes such as gender and age. Female and younger users were more likely to adopt electronic health systems [15]. In this study, all participants were females and relatively young.

The participants discussed issues related to human rights. According to them, JSANDS protects patients’ privacy to a high degree. However, they reported occasional possible violation of human rights when unauthorized access become possible. One limitation raised by the participants, which could lead to unauthorized access to patient records, was that the system would remain open in case the user forget to log out. To ensure patients’ autonomy, all of their records must be maintained confidential and private [16]. Patient records should be kept private from unauthorized use [16]. In their report of the second global survey on eHealth that addressed patient privacy in EHRs, the WHO stated that only one third of the countries globally reported having privacy protection legislation in place. Such legislations are mainly found in developed countries [17]. While privacy protection is an important feature that needs to be addressed in any electronic system, other legal and ethical issues need to be an integral part of any health care system infrastructure. Ensuring system safety necessitates a shared responsibility between developers and organizations to create policies that control the design, development and the use of any digital solutions [18].

Other limitations reported by users included some technical problems and poor internet connections, and occasional loss of previously recorded data. Similar to our findings, these issues were suggested by a previous research as potential negative influences of the electronic health record use [19]. In addition, a previous Jordanian study also documented similar technical problems including poor internet connection, system crashes and freezes as being a threat to patient safety [11]. While these are related to technical issues rather than system quality issues, organizations need to ensure appropriate infrastructure to ensure proper system performance.

Moreover, participants expressed concerns about failure to modify data entries after a period of 28 days, and inability to manually record the cause of death in case if the main cause of death is not listed. Nurses in a previous study have suggested that using both a ready-made template and a free-text approach allow for more complete and comprehensive data, and therefore better quality of patient care [11].

The JSANDS users described some instances where using the system had improved their communication skills with the patients and enhanced their sense of responsibility toward numbers of deaths. Previous research documented opposing effects of the use of EHRs on clinician-patient interactions; it may facilitate or inhibit their interaction by diverting the clinician attention away from the patient [16]. In addition, similar to our findings, the use of EHR was reported to enhance the clinical reasoning skills of health care providers [13, 16].

As discussed earlier, the data generated by the JSANDS is crucial for minimizing stillbirths and neonatal deaths in Jordan since it allows the use of data to take early and appropriate actions. If the Ministry of Health consider adopting this system, there is a need for regular upgrading and training for users in order to reduce possible errors and to produce complete and worthy data. Many factors may influence the adoption and sustainability of any electronic health system such as positive users’ attitude, skills base, and good leadership and communication [20]. In addition, similar to our results, the need for ongoing training and the availability of financial incentives were suggested to have positive influence on electronic system adoption [15].

The results of this study has potential implications for further development of JSANDS. A surveillance system that is easy to use and able to track stillbirths and neonatal deaths and their causes is crucial for a successful implementation. Continuous monitoring and evaluation should be in place to enhance the sustainability and the effectiveness of any implemented system including JSANDS. The findings of this study are important for enhancing JSANDS to be scaled up to other hospitals in Jordan.

### Limitations

As with other qualitative research, the study findings would be affected by researchers’ values, as the researcher is the main instrument in qualitative studies. This highlights the need to reflect on our roles as an active participant in the research process [21], and to acknowledge our influence on the research findings. The research team tried to acknowledge their subjectivity and strived to maintain an open-minded approach through continuous self-evaluation [22]. The researchers who were involved in the data collection and data analysis processes kept research diaries on their personal reflections on methodological issues such as sampling, interviews, and analysis. In addition, to ensure credibility of the data, member-check was established, which involved asking some participants to review the preliminary analysis of their viewpoints to determine whether they recognize that the findings reflect their own experiences.

Furthermore, it should be noted that this paper only presents data about the focal points’ experiences, who were all senior nurses. This may constitute a threat of bias and unknot representative of the population in question. Experiences of other users including junior staff member could be different, and therefore, future studies that include a wide range of the system users are recommended.

Finally, this is a descriptive qualitative study, and we only present the users’ perceptions. As with other qualitative research, it is possible that there was additional information about the system that was not communicated during the FGs. We also cannot guarantee that all the participants’ perceptions that were communicated during the FGs were accurate. In order to be able to generalize the results, future research to assess the system in terms of these variables is required.

## Conclusions

Findings of this study have suggested that the JSANDS hold promise for use in hospitals as an electronic stillbirths and neonatal deaths surveillance system. Data generated by this system has many potential uses including, but not limited to, planning and implementing programs to limit perinatal deaths, identifying high risk and marginalized populations, measuring the burden of stillbirths and neonatal mortality, prioritizing health resources, and offering a basis data for epidemiologic research. In addition, the system helps to reveal the causes of these deaths, which is important for determining appropriate actions. The depth of data generated by the JSANDS is crucial for describing changes in stillbirths and neonatal mortality and identifying health care priorities. This study provided essential information that may be used for further development of the JSANDS system. While the users acknowledged many positive features of the JSANDS, they also reported some limitations, which if addressed may enhance the system usability and sustainability.

## Supplementary Information

**Additional file 1: Appendix 1:** Question guide.

## Data Availability

The datasets used and/or analysed during the current study are available from the corresponding author on reasonable request.

## Abbreviations

JSANDS: Jordan Stillbirth and Neonatal Death Surveillance
ICD-PM: International Classification of Diseases-Perinatal Mortality
