# Supplementary material for: The usability of Jordan stillbirths and neonatal deaths surveillance (JSANDS) system: results of focus group discussions
Source: Arch Public Health. 2021 Mar 7;79:29. doi: 10.1186/s13690-021-00551-1 (PMC7937354; doi:10.1186/s13690-021-00551-1)
Supplement: Supplementary file 1 — Additional file 1: Appendix 1: Question guide. [file 13690_2021_551_MOESM1_ESM.docx]

# **Appendix 1:** Question guide

**Opening question:**

- How would you describe your experience with using the JSANDS system?

**Follow-up Questions**

- Do you think this system has achieved its goals?
- Can you please address the need to continue and/or modify the JSANDS?
- Do you believe the JSANDS is simple and easy to use by healthcare professionals? Do you think it needs well-trained person to be able to use it? Can you please share your experience with using the system?
- How flexible do you believe the JSANDS is? Can you please share an example from your use of this system?
- Do you think that the JSANDS is well-accepted by healthcare professionals (nurses, midwives, resident doctors, senior doctors)? Or do they reject it and resist using it?
- To what extent do you think the data generated from the JSANDS is sensitive and accurate?
- How do you use the information generated from the system?
- Can you please describe the people that have used the data to make decisions and take actions?
- What are other anticipated uses of the data?
- How to improve the system?
- Do you have any suggestions or recommendations about the system?
- What are the expected long-term outcomes?
- What can be done to further enhance sustainability of the project?
- Do you think the system is cost effective? Explain.
- Do you plan to continue using JSANDS once the project is completed? Explain
- Do you expect the project to be adopted by other hospitals nationally and internationally? Explain
- What is the average time needed to register a birth and/or a death? And do you think it can be incorporated within your time schedule during a working day? Ir is it a burden for you and your colleagues?
- What decision-making or public health actions have been taken as result of the data? OR: Can you please tell us the level of usefulness of the JSANDS? In other words: describe the actions taken as a result of the data generated from the JSANDS?
- Does the JSANDS system have any impact on your work? How?
- Do you believe that acknowledging and reporting a neonatal death through the JSANDS strengthens the position and rights of the living by acknowledging that every child is a human being from the moment of birth?
- How the JSANDS enhanced female HCPs (nurses and midwives)
- What are the needs of the users that were not anticipated by the system developers?
- What do you think are the strengths of the JSANDS system?
- What do you think the weaknesses/ limitations of the JSANDS system?
- Any suggestion or recommendations to improve the JSANDS and its performance**.**
- How do you think that JSANDS helped in identifying misfortunate areas?
- How do you think that JSANDS helped in identifying marginalized/ disadvantaged population?
- How do you think that JSANDS helped in identifying high risk population?
- Do you think JSANDS helped ensuring human rights through the registration system? How?
- What do you think about privacy and security of the data arising from the JSANDS?
- Who do you think should have access to these data?
- How do you think this data could be used/shared?
- In your opinion, how Covid-19 has affected births/delivery process in your hospital.
- In your opinion, how Covid-19 has affected the stillbirths and neonatal deaths in your hospital.
